# Estimating the Worldwide Extent of Illegal Fishing

**DOI:** 10.1371/journal.pone.0004570

**Published:** 2009-02-25

**Authors:** David J. Agnew, John Pearce, Ganapathiraju Pramod, Tom Peatman, Reg Watson, John R. Beddington, Tony J. Pitcher

**Affiliations:** 1 Division of Biology, Imperial College London, London, United Kingdom; 2 MRAG Ltd, London, United Kingdom; 3 Fisheries Centre, University of British Columbia, Vancouver, British Columbia, Canada; 4 United Kingdom Government Office for Science, London, United Kingdom; University of California San Diego, United States of America

## Abstract

Illegal and unreported fishing contributes to overexploitation of fish stocks and is a hindrance to the recovery of fish populations and ecosystems. This study is the first to undertake a world-wide analysis of illegal and unreported fishing. Reviewing the situation in 54 countries and on the high seas, we estimate that lower and upper estimates of the total value of current illegal and unreported fishing losses worldwide are between $10 bn and $23.5 bn annually, representing between 11 and 26 million tonnes. Our data are of sufficient resolution to detect regional differences in the level and trend of illegal fishing over the last 20 years, and we can report a significant correlation between governance and the level of illegal fishing. Developing countries are most at risk from illegal fishing, with total estimated catches in West Africa being 40% higher than reported catches. Such levels of exploitation severely hamper the sustainable management of marine ecosystems. Although there have been some successes in reducing the level of illegal fishing in some areas, these developments are relatively recent and follow growing international focus on the problem. This paper provides the baseline against which successful action to curb illegal fishing can be judged.

## Introduction

It is widely accepted that there is a severe problem with future global food security. Driven by substantial world population growth, demand for fish protein continues to increase, but a large number of the world's fish stocks are currently depleted and therefore not capable of producing their maximum sustainable yield [1]. Illegal and unreported fishing (in this paper taken to include illegal and unreported catches but to exclude discards and artisanal unregulated catches) prejudices the managed recovery of the world's oceans from severe fish depletions [2]–[4]. It is reported to lead to a loss of many billions of dollars of annual economic benefits [5], [6], creates significant environmental damage through the use of unsustainable fishing practices [7] and has wider consequences for food supply [8].

Estimating the level of illegal fishing is, by its very nature, extremely difficult and has not previously been attempted on a global scale. Fishing vessels, especially those fishing in high seas waters and under third party access agreements to EEZ waters (Exclusive Economic Zones, which can extend up to 200 nm from the coast), are highly mobile. Although there are a number of studies of the level of IUU (Illegal, Unreported and Unregulated) fishing in individual fisheries (both EEZs and high seas) [3], [9]–[16], only two studies have attempted to estimate the impacts of IUU over a whole region [5], [6]. In this paper we set out, for the first time, a detailed study which arrives at global estimates of current and historical illegal and unreported catches.

## Results

The term “Illegal, Unreported and Unregulated” (IUU) fishing can cover a wide range of issues [see discussion in 13], [17]–[19]. We confined our analysis to illegal and unreported catches (IU), namely those taken within an EEZ which are both illegal and retained, and which are usually unreported, and all unreported catches taken in high seas waters subject to a Regional Fisheries Management Organisation's (RFMO) jurisdiction. We excluded discards and unregulated artisanal catches, which will be analysed in a future publication. With illegal and unreported catches rents are captured by illegal fishermen but lost to legitimate fishermen and management authorities. Note that the word “landings” is often used to distinguish catches that are retained from catches that are discarded. For simplicity, and to avoid confusion with the suggestion that fish are necessarily landed in the country in whose waters they are caught, we use the word catches here to mean catches that are retained and discards to mean catches that are discarded.

In total, 54 EEZs and 15 high seas regions were analysed, providing an estimate of global illegal and unreported catch for 292 case study fisheries which comprise 46% of the reported total world marine fish catch. All data sources were combined to provide upper and lower estimates of IU for each fishery. The total catch of case study and non-case study fish from the EEZs and high seas regions analysed comprises 75% of global catch.

There were significant differences in the level of illegal and unreported catch and the trends in those catches between regions. The level of IU was highest in the Eastern Central Atlantic (Area 34) and lowest in the Southwest Pacific (Area 81) (Table 1). Since the 1990s we estimate that the level of IU has declined in 11 areas and increased in 5 (Table 2). We estimate that the overall loss from our studied fisheries is 13–31% (lower and upper estimates) with a mean of 18%, and that this was worth some $5-11 bn in 2003.

**Table 1:** Summary of regional estimates of illegal fishing, averaged over 2000–2003.

Region	Reported catch of case study species	Catch of case study species as a percentage of total regional catch	Lower estimate of illegal catch (t)	Upper estimate of illegal catch (t)	Lower estimate of value (US$m)	Upper estimate of value (US$m)
Northwest Atlantic	557,147	25%	22,325	82,266	20	74
Northeast Atlantic	6,677,607	60%	364.908	842.467	328	758
Western Central Atlantic	390,942	22%	21,745	58,514	20	53
Eastern Central Atlantic	1,154,586	32%	294,089	562,169	265	506
Southwest Atlantic	1,403,601	65%	227,865	673,712	205	606
Southeast Atlantic	1,351,635	79%	52,972	139,392	48	125
Western Indian	2,165,792	52%	229,285	559,942	206	504
Eastern Indian	2,263,158	44%	467,865	970,589	421	874
Northwest Pacific	7,358,470	32%	1,325,763	3,505,600	1,193	3,155
Northeast Pacific	196,587	7%	2,326	8,449	2	8
Western Central Pacific	3,740,192	36%	785,897	1,729,588	707	1,557
Eastern Central Pacific	1,374,062	73%	129,772	278,450	117	251
Southwest Pacific	451,677	61%	5,227	32,848	5	30
Southeast Pacific	9,799,047	73%	1,197,547	2,567,890	1,078	2,311
Antarctic	136654	100%	9593	9593	9	9
**Total**	**39,021,155**	**46%**	**5,140,928**	**12,040,052**	**4,627**	**10,836**

**Table 2:** Trends in regional estimates of illegal fishing, averaged over 5 year periods 1980–2003.

Region	1980–1984	1985–1989	1990–1994	1995–1999	2000–2003
Northwest Atlantic	26%	19%	39%	15%	9%
Northeast Atlantic	10%	10%	12%	11%	9%
Western Central Atlantic	16%	14%	14%	11%	10%
Eastern Central Atlantic	31%	38%	40%	34%	37%
Southwest Atlantic	15%	18%	24%	34%	32%
Southeast Atlantic	21%	25%	12%	10%	7%
Western Indian	31%	24%	27%	25%	18%
Eastern Indian	24%	29%	30%	33%	32%
Northwest Pacific	16%	15%	23%	27%	33%
Northeast Pacific	39%	39%	7%	3%	3%
Western Central Pacific	38%	37%	37%	36%	34%
Eastern Central Pacific	20%	17%	13%	14%	15%
Southwest Pacific	10%	9%	7%	7%	4%
Southeast Pacific	22%	21%	24%	23%	19%
Antarctic	0%	0%	2%	15%	7%
**Average**	**21%**	**21%**	**21%**	**20%**	**18%**

The figure given is the mid-point between the lower and upper estimates of illegal and unreported catch in the case study species, expressed as a percentage of reported catch of case study species.

Regional trends reveal issues related to the quality of fishery management. In the Northeast Atlantic, reasonable estimates of the level of illegal fishing are available from various reports and assessments conducted by the International Council for the Exploration of the Sea, ICES [20]. These indicate that as pressure on stocks increased following the end of the ‘gadoid outburst’ (exceptional recruitment from cod family fish between the mid 1970s to late 1980s) the level of illegal and unreported catch increased, and has only recently improved. The decline in IU that we show in the Western Central Atlantic is due to a reduction in the upper bound of uncertainty over unreported tuna catches. The introduction by the International Commission for the Conservation of Atlantic Tunas (ICCAT) of statistical document schemes required for trade in tuna has significantly decreased the amount of unreported tuna catch in the Central Atlantic [9]. In the Eastern Central Atlantic there appears to have been a steady increase in illegal fishing, which is at a much higher level than in the western central Atlantic. This is a large area, covering many states with a wide variety of fisheries and governance (Morocco to Angola), some of which, such as Guinea, Sierra Leone and Liberia suffered increasing illegal catches as a result of internal strife in the 1990s. We have increasing uncertainty about the level of illegal fishing in the Soutwest Atlantic from the mid-1990s, but overall the proportion of illegal catch appears to have increased at this time, once again in response to declining resource status. In contrast, the exclusion of foreign vessels from Exclusive Economic Zones in the Southeast Atlantic, and the imposition of national control in Southeast Atlantic coastal states from the late 1980s, led to a marked reduction in illegal fishing at that time.

The decline of illegal fishing in the Western Indian Ocean reflects gradually increasing control over time by coastal states, particularly those in the extreme north and countries of the Southern African Development Community, and a reduction in the unreported catch estimated by the Indian Ocean Tuna Commission (IOTC). The increase in estimated illegal fishing in the Northwest Pacific is almost entirely due to the influence of China and Russia, since estimates of illegal catch in other states in the area are relatively small. However, the confidence in this estimate is not as good as for other estimates in this analysis, which is reflected in an increase in uncertainty in this region. Northeast Pacific illegal catch is currently estimated to be low and to have steadily declined over recent years, but, surprisingly, we were unable to obtain good estimates from the USA. Western Central Pacific data include coastal states of the western Pacific seaboard, where the information available to us suggests that a relatively high level of illegal and unreported catch has been present with little change over the years. For instance, in Indonesia a huge amount of unreported catch (over 1.5 million tonnes annually) has recently been revealed by an FAO study of the Arafura Sea, much of this illegal [21]. In the Eastern and Southeastern Pacific a similar situation of low change exists, but with a much lower estimated proportion of illegal fishing. In the Southwest Pacific increasing control by coastal states has led to a significant reduction in illegal fishing over the last 20 years.

Finally, in the Antarctic, the only illegal fishing issue is unregulated and unreported fishing for toothfish, which peaked in 1996 and has since significantly reduced.

**Figure 1:**
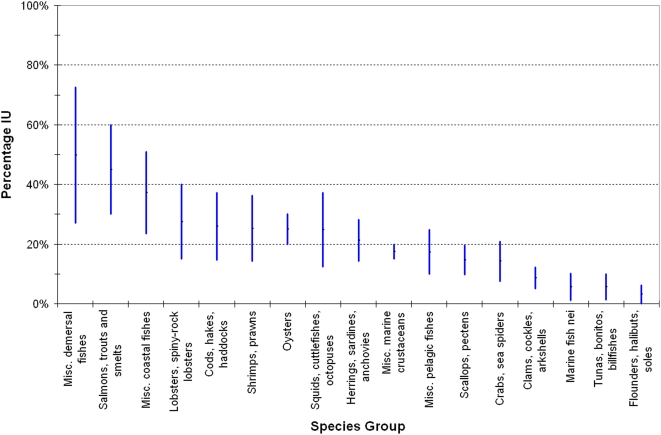
Illegal and unreported catch, expressed as a percentage of reported catch, by species group 2000–2003. Upper and lower bounds are given.

As would be expected, the highest levels of illegal fishing are associated with high value demersal fish, lobsters and shrimps/prawns (Figure 1). It is somewhat surprising at first glance that the proportion of illegal catch is low for tunas. The reason for this is that most tuna catches are taken within the areas of RFMOs where the small amounts of unreported fishing are generally associated with large volume catches (for instance of yellowfin and bigeye tuna) and in some regions (e.g., the Inter-American Tropical Tuna Commission and the IOTC) unreported catches of tunas are now very small.

Taking the total estimated value of illegal catch losses and raising by the proportion of the total world catch analysed in this paper, lower and upper estimates of the total value of current illegal and unreported fishing losses worldwide are between $10 bn and $23.5 bn annually, representing between 11 and 26 million tonnes. The estimates previously made by MRAG [6] ($9 bn) and Pauly et al. [5] ($25 bn) fall at either end of this range. Estimates of losses from illegal logging are of the same magnitude, roughly 10% of world timber trade with illegal products worth at least $15 bn a year [22].

**Figure 2:**
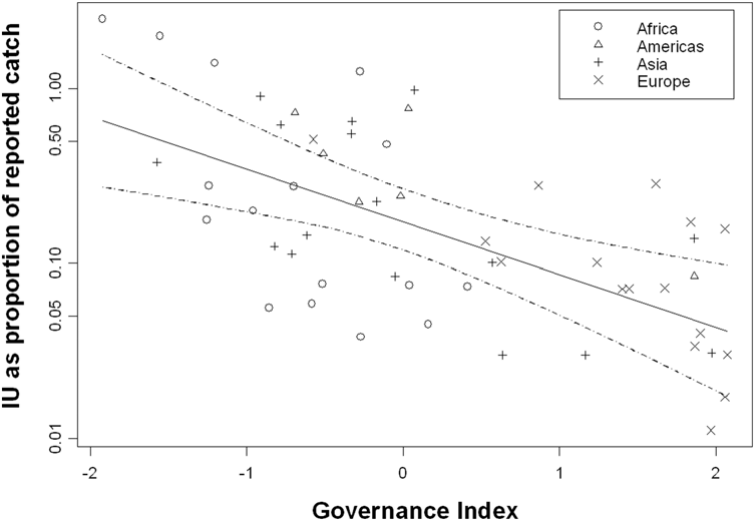
Relationship between the amount of illegal fishing (expressed as a proportion of the reported catch that is additionally taken as illegal and unreported catch) and an average of four World Bank indices of governance (Government Effectiveness, Regulatory Quality, Rule of Law and Control of Corruption, measured in 2003; *23*). Although there is a significant linear relationship between governance and the proportion of IU, the log-linear relationship shown above is a better fit to the data and has R^2^ = 0.4081, p<0.001 with 53 degrees of freedom. The broken lines are 95% confidence intervals.

Since there are strong economic drivers for illegal fishing [3], [4] and it occurs in situations of poor fisheries management and control [2], [6], we might expect that the level of illegal fishing would be related to fish price, governance and indicators of the control problem, such as the area of a country's EEZ and the number of patrol vessels at its disposal. We found no significant relationship between illegal fishing and the price of fish or the size of the EEZ or the fishery in our study, but we did find a significant relationship with World Bank governance indicators measured in 2003 [23], which was strongest with the log of illegal fishing level. This relationship was significant for the whole dataset (Figure 2) (R^2^ 0.400, p<0.001, n = 54), for Africa, Europe and Asia separately (R^2^ 0.393, 0.375, 0.429, p<0.01, 0.05 and 0.01 respectively, n = 16 in each case), and with different indicators of governance such as the Corruption Perceptions index [24] (R^2^ 0.371, p<0.001, n = 50).

## Discussion

This is the first time, to our knowledge, that a significant relationship has been demonstrated on a global scale between the level of illegal and unreported fishing and indices of governance, and it points to the benefits of improving governance. This is not to say that developing countries with poor governance records are necessarily to blame for illegal fishing, but that they are more vulnerable to illegal activities, conducted by both their own fishers and vessels from distant water fishing nations. In Africa, for instance, many coastal states licence vessels from distant water fishing nations such as China, Taiwan, Korea, the EU and Russia to fish in their waters, and there is a significant illegal fishing problem from many of these vessels [6]. This represents a failure of control on behalf of the flag state as well as the coastal state. Furthermore, many vessels engaged in IUU activities are registered with so-called ‘flag of convenience’ states, and whilst these are mostly developing countries the vessels themselves are usually owned and operated by developed country companies [25].

On a world scale, poor performance in the control of illegal fishing is pervasive. In a recent review [26] over half of the countries (30/53 top fishing countries) assessed for compliance with illegal and unreported fishing in the FAO (UN) Code of Conduct for Responsible Fisheries [27], [28; Article 6.10; Articles 7.6.1, 7.7.1, 7.7.5, and 7.8.1] were awarded fail grades (less than 4/10). Only a quarter (16/53) were rated as ‘passable’ (6/10 or more). Moreover, implementation of ecosystem-based management requires control of illegal fishing, and here again almost half of the countries surveyed failed (16/33), while only two received a ‘good’ rating [29].

Illegal and unreported fishing can have very significant effects on stocks. For instance, unreported catches of bluefin tuna from the Mediterranean (estimated by the International Commission for the Conservation of Atlantic Tunas to have been 19,400 t in 2006 and 28,600 in 2007; 30) have significantly contributed to the rapid decline in the stock, and a failure by the European Union to control unreported catches led to a failure to generate any recovery in North Sea cod until very recently [31]. There is a correspondence between our regional estimates of illegal and unreported fishing and the number of depleted stocks in those regions. For instance out of 53 demersal stocks recognised in the eastern central Atlantic, 32 of which could be assessed, 60% were overexploited in the early 2000s [32] compared to 30% of EU stocks and 15% of New Zealand stocks [2]. Thus out of these three areas those with the highest and lowest proportion of depleted stocks also had the highest and lowest levels of illegal fishing (Table 1). This may be both because illegal and unreported fishing is contributing to overexploitation of stocks, and because the general management of stocks (including the quality of research, for instance) is likely to be better in areas of higher quality governance. Illegal fishing in regions with poor governance has often been linked to organised crime [25], [33], but where fish have a high value, this can be an issue even in countries with good governance [34].

Illegal fishing creates significant collateral damage to ecosystems. Illegal fishing, by its very nature, does not respect national and international actions designed to reduce bycatch and mitigate the incidental mortality of marine animals such as sharks, turtles, birds and mammals. Such practices are common: examples are illegal fishing in marine reserves in west Africa [6], [7] and the bycatch of albatross in illegal and unreported longline and gillnet operations in the Antarctic [11], [35]. Only a solution of the illegal fishing problem will generate the compliance with these wider ecosystem management measures. Moreover, as part of the move to explore ecosystem-based management, estimates of unreported catches have proved to be necessary to balance ecosystem models [36]. Where unreported extraction of fish from major stocks is not included this can bias both single species stock assessment and ecosystem-based analyses in a dangerous direction of allowing more fishing than would otherwise be thought sustainable.

Clearly some progress has been made in some areas over the last decade; our study identifies reductions in illegal fishing in 11 areas since the early 1990s and indeed this trend has continued in the years since 2003. The worst period for illegal and unreported fishing world wide appears to have been the mid-1990s, driven by a combination of factors: a growing world demand for fish and significant overcapacity of the world's fishing fleet set against increasing limitation of access to distant water fishing nations and a lack of new or alternative fishing opportunities [1], [37].

The solutions most often proposed to eliminate illegal fishing are associated with increased governance and the rule of law - increased cooperation between regional management authorities in management and control activities, increased capacity to undertake surveillance and enforcement of port state control [38], and other means of reducing the economic incentives to engage in IUU fishing, such as increased sanctions and trade measures [4], [39]. Recent successes emphasise this. There has been a significant reduction in illegal and unreported catches of cod from 50% to 20% of the reported catch in the Barents Sea following cooperative port state controls implemented by the states party to the Northeast Atlantic Fisheries Commission [40] and in the Antarctic IUU catches have been reduced from 33,700 t in 1996/97 to 3,600 t in 2006/07 through cooperative international and state action [36].

These activities are encouraging, but set in the context of burgeoning demand for food and particularly protein, there will continue to be enormous pressure on fish stocks over the next 50 years and it is essential that the international community address effectively the large illegal and unreported catch of fish reported in this paper. Given the recent change in political will to tackle the issue of illegal fishing, [e.g. 38], further improvements might be expected to come from legally mandating compliance with the FAO (UN) Code of Conduct for Responsible Fisheries [25], which would provide countries with an international legal basis for economic and other sanctions that discourage illegal fishing. Some countries already include some of the provisions of the Code of Conduct in their national legislation (for example in Australia, South Africa, Norway, Namibia, Malaysia). Others, such as the EU, are now proposing to implement much stronger import controls and sanctions to restrict trade in IUU fish. This paper provides the baseline against which action to curb illegal fishing can be judged.

## Methods

We used the “anchor points and influence table” approach of Pitcher et al. [13] which employs detailed reports (from published scientific literature and in-country specialist studies) to establish point estimates and upper and lower bounds of the level of illegal fishing in different fisheries, and identifies changes to these levels over time based on historical data or likely trends based on known changes in management regime. In the source studies a number of different methods have been used to estimate the level of illegal fishing, including surveillance data, trade data, stock assessments based on fishery-independent (survey) data and expert opinion [7]. Some of these methods deliver a point estimate of the level of illegal fishing, some deliver statistical estimates with confidence intervals, and some deliver upper and lower bounds. We took the approach that, when trying to integrate the results of these various estimation methods with their differing levels of reliability, using the extreme upper and lower limits produced less variation than trying to make a point estimate. Where it was available the point estimate was used to set initial bounds for a percentage figure, but we used expert opinion to guide the upper and lower limits, and did not treat them as two point estimates.

Countries were selected based on the volume (tonnage) of catches reported to have been taken in their EEZ in order of magnitude (i.e., their importance as fishing areas). A few additional countries with smaller catches (5 in all, 1.4% of world catch) were included because of their importance in understanding the distribution of illegal fishing. All Regional Fishery Management Organisations (RFMO) were examined. Because data were required by EEZ rather than FAO area, and in order to keep catches consistent with FAO totals, catch data for each EEZ selected and for each high seas FAO region were extracted from the Sea Around Us project database of estimated catches (41; www.seaaroundus.org). The Sea Around Us project has attributed FAO catches reported to EEZs and High Seas by means of a geospatial algorithm [41]. Within each study, catches and IUU fishing (discarded and illegal assembled separately) of the four highest volume (i.e., tonnage caught) species were estimated from the source studies (seven countries only had three species listed). This led to 292 separate fishery estimations per year. Percentage trends for IUU in each five year block were multiplied by the annual reported catch data to form overall annual estimates, separated into illegal catches and discards (including other unreported fish catch such as recreational and legal but unreported artisanal catches).

EEZs selected for the main time-trend analysis were: Angola, Argentina, Australia, Bangladesh, Brazil, Canada, China, Denmark, Ecuador, Faeroe Islands, France, Germany, Ghana, Iceland, India, Indonesia, Iran, Ireland, Italy, Japan, Republic of Korea, Latvia, Malaysia, Mexico, Morocco, Mozambique, Myanmar, Namibia, Netherlands, New Zealand, Nigeria, Norway, Pakistan, Peru, Philippines, Poland, Portugal, Russia, Senegal, South Africa, Spain, Sri Lanka, Sweden, Thailand, United Kingdom, Tanzania, Yemen. RFMOs analysed for time trends of illegal and unreported catches were: Northwest Atlantic Fisheries organisation (NAFO), Northeast Atlantic Fisheries Commission (NEAFC), the Commission for the Conservation of Antarctic Marine Living Resources (CCAMLR), the Inter American Tropical Tuna Commission (IATTC), the International Commission for the Conservation of Atlantic Tuna (ICCAT), the Indian Ocean Tuna Commission (IOTC), the Western and Central Pacific Fisheries Commission (WCPFC) and the Commission for the Conservation of Southern Bluefin Tuna (CCSBT). Our primary data sources were several key composite studies [6], [20], [42]–[44], supplemented by country-specific studies [15], [19], [33], [45]–[86].

In order to estimate the global level of illegal and unreported catches (IU) a single estimate for the price of a tonne of fish each year was used. The price data used were those reported by FAO [87].

For some countries a historical time series of estimates of IU could not be derived from available data sources, although data were available from the early 2000s from MRAG [6] (Guinea, Kenya, Liberia, Papa New Guinea, Seychelles, Sierra Leone, Somalia). While they have been included in the analysis of governance relating to illegal fishing, these data do not contribute to the table showing the trends in illegal catch over time by region.

For each case study and species the analysis generated the following

T_cy_ total reported tonnage of all wild fish caught in the case study EEZ/RFMO area *c* in year *y*
t_csy_ reported tonnage of fishery *s* in case study *c*
U_csy_ upper bound estimate of illegal catch
L_csy_ lower bound estimate of illegal catch

The estimate of illegal catch as a proportion of reported catch for a case study and year was calculated as 
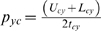
 where 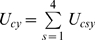, and so on.

Regional estimates were developed by combining the high seas estimates along with EEZ estimates within that region. Where an EEZ was covered by a number of different FAO regions, these EEZs were where possible divided into two separate estimates (e.g., the estimate for the Russian EEZ was broken down by for the Atlantic and Pacific catches, and Canada and Mexico for west and east coasts). If this was not possible, the data reported by FAO area and recorded in the FAO FISHSTAT database were used to determine the approximate percentage of catches taken in each area and the estimates distributed uniformly with reported catches (e.g., South Africa, Australia and USA).

The confidence intervals shown in Figure 2 were created by estimating the confidence intervals for 1000 simulated datasets where, for each country, the level of IU was sampled from a uniform distribution defined by our upper and lower estimates of IU, and governance was sampled from a gaussian distribution with mean and standard deviation as presented in Lambsdorff [24]. The confidence intervals plotted in the paper are the maximum upper 95% and minimum lower 95% limits from the 1000 simulations.
